# Identifying the Growth Status of Hydroponic Lettuce Based on YOLO-EfficientNet

**DOI:** 10.3390/plants13030372

**Published:** 2024-01-26

**Authors:** Yidong Wang, Mingge Wu, Yunde Shen

**Affiliations:** College of Mechanical and Electrical Engineering, Wenzhou University, Wenzhou 325035, China; 21461440073@stu.wzu.edu.cn (Y.W.); wmg7810@wzu.edu.cn (M.W.)

**Keywords:** hydroponic lettuce, growth status, object detection, object classification

## Abstract

Hydroponic lettuce was prone to pest and disease problems after transplantation. Manual identification of the current growth status of each hydroponic lettuce not only consumed time and was prone to errors but also failed to meet the requirements of high-quality and efficient lettuce cultivation. In response to this issue, this paper proposed a method called YOLO-EfficientNet for identifying the growth status of hydroponic lettuce. Firstly, the video data of hydroponic lettuce were processed to obtain individual frame images. And 2240 images were selected from these frames as the image dataset A. Secondly, the YOLO-v8n object detection model was trained using image dataset A to detect the position of each hydroponic lettuce in the video data. After selecting the targets based on the predicted bounding boxes, 12,000 individual lettuce images were obtained by cropping, which served as image dataset B. Finally, the EfficientNet-v2s object classification model was trained using image dataset B to identify three growth statuses (Healthy, Diseases, and Pests) of hydroponic lettuce. The results showed that, after training image dataset A using the YOLO-v8n model, the accuracy and recall were consistently around 99%. After training image dataset B using the EfficientNet-v2s model, it achieved excellent scores of 95.78 for Val-acc, 94.68 for Test-acc, 96.02 for Recall, 96.32 for Precision, and 96.18 for F1-score. Thus, the method proposed in this paper had potential in the agricultural application of identifying and classifying the growth status in hydroponic lettuce.

## 1. Introduction

Lettuce is considered a vegetable with high economic value, so it is widely cultivated within China. China’s lettuce cultivation area accounts for more than half (51.6%) of the global cultivation area. Furthermore, China is the largest lettuce producer in the world, with a total output greater than the sum of all other countries (India, USA, Spain, Italy, Turkey, Japan, Mexico, and other countries). Its share of the global harvest is 56.4% [1]. In terms of its nutritional value, lettuce has the characteristics of low calorie, low fat, and low sodium content, and it also contains a large amount of carotene, antioxidants, vitamins B1, B6, E, and C, as well as rich dietary fiber and various trace elements [2,3]. The currently promoted method of cultivating lettuce is through hydroponics, mainly because this method has the advantages of high production efficiency and minimal environmental pollution [4,5]. However, there are some challenges in growing hydroponic lettuce due to the large scale of planting, the complexity of the hydroponic environment, and the growth characteristics of lettuce itself. One of them is that lettuce is fragile and vulnerable to diseases and pest infestations during the transplanting and growth process [6,7]. In order to avoid further spread of pests and diseases and to ensure the growth quality and yield of hydroponic lettuce, manual inspection and replacement of problematic lettuce becomes a necessary measure.

The traditional identification of the growth status in hydroponic lettuce mainly relies on manual identification by experts and growers, which faces many problems in practical production management such as being time-consuming, laborious, and inefficient [8]. With the application of modern information technologies in agriculture, such as big data, artificial intelligence, and cloud computing, intelligent identification methods have gradually been applied to monitor the growth status and identify pests and diseases of various crops [9]. Currently, intelligent recognition methods mainly include machine learning techniques and deep learning techniques. Machine learning techniques can extract relevant features of growth status by performing mathematical operations and image processing on plant growth images and then train classifiers to achieve recognition of growth status. For example, Pantazi et al. [10] applied a support vector machine (SVM) algorithm to create three one-class classifiers for detecting powdery mildew, downy mildew, and black rot on the leaves of 46 crops, achieving a classification accuracy of 95.0%. Lu et al. [11] used three classification models, sequential discriminant analysis (SDA), fisher discriminant analysis (FDA), and k-nearest neighbors (KNN) algorithm, to classify early-stage anthracnose crown rot infection on strawberry leaves. The average classification accuracies of SDA, FDA, and KNN were 71.3%, 70.5%, and 73.6%, respectively. Xie et al. [12] defined KNN sorting features and used hyperspectral imaging to classify healthy and gray moldy tomato leaves with 97.2% classification accuracy. Sun et al. [13] combined simple linear iterative clustering (SLIC) with SVM to detect anthracnose and brown blight of tea tree in a complex background with 96.8% classification accuracy. However, the establishment of these recognition models is challenging as it requires manual adjustment of numerous model parameters and the model training process is prone to overfitting.

Deep learning technology is indeed an important branch in the field of machine learning. It simulates the functioning of the human brain by constructing multi-layer neural network models, enabling automated data analysis and feature extraction such as color, texture, and shape [14,15]. In deep learning, convolutional neural networks (CNN) are widely used for image processing and visual-related tasks. Athanikar and Badar [16] applied a CNN to classify potato leaf images as healthy or sick. Their experimental results show that CNN can effectively detect disease spots and classify specific disease types with 92% accuracy. Sladojevic et al. [17] proposed a CNN-based deep neural network model that can recognize 13 different plant diseases from a collection of images of healthy and diseased leaves and is able to distinguish plant leaves from their surrounding environment. The recognition accuracy of the model ranges from 91% to 98% with an average accuracy of 96.3%. Mohanty et al. [18] tested classic network models AlexNet and GoogLeNet using a training set of 14 crop varieties and 26 types of pests and diseases in the PlantVillage database to predict crop pests and diseases for plant leaf images. Too et al. [19] fine-tuned and compared various classic convolutional neural networks, such as VGG-16, Inception-V4, DenseNets-121, and ResNet-50, using data from 14 plants and 38 categories of pests and diseases from the PlantVillage dataset. The experimental results showed that the accuracy of DenseNets consistently improved without overfitting as the number of iterations increased. Moreover, DenseNets achieved a testing accuracy of 99.75% with few parameters and a short training time. Therefore, deep learning may have more advantages in crop variety identification.

Based on the above research status, this paper focused on hydroponic lettuce as the research subject and proposed a hydroponic lettuce disease and pest recognition method using YOLO-EfficientNet. The paper used the YOLO-v8n model to select and crop target objects from a video dataset of hydroponic lettuce and then utilized the EfficientNet-v2s model to recognize and classify three different growth stages of the cropped images. This proposed approach provided a new perspective and method for identifying disease and pest damage in hydroponic lettuce, and it could serve as a reference for further research in related fields.

## 2. Materials and Methods

### 2.1. Experimental Field

**Figure 1:**
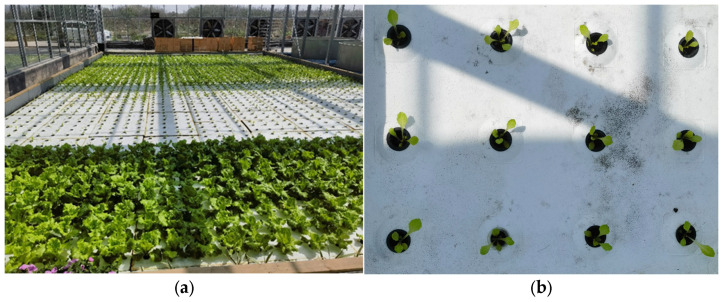
Experimental environment of hydroponic lettuce. (**a**) Experimental base for growing hydroponic lettuce. (**b**) Lettuce seedlings planted on holed foam boards.

The variety of hydroponic lettuce used in this article was Romaine lettuce and the seed supplier was China Vegetable Planting Industry Technology Co., Ltd. (Beijing, China). The hydroponic lettuce growth experiment was conducted in a greenhouse at Yangdu Research and Innovation Base of Zhejiang Academy of Agricultural Sciences during March 2023–June 2023 (latitude 30°43′83″ N, longitude 120°41′58″ E), as shown in Figure 1a. During the transplanting process, hydroponic lettuce seedlings were selected with a seedling age of approximately two weeks and a leaf count of 3 to 5 leaves. The seedlings were planted on holed foam boards, with each board accommodating 12 hydroponic lettuce seedlings (as shown in Figure 1b), and the spacing between each seedling was 0.3 m. In order to facilitate the collection of image data on the growth status of hydroponic lettuce after transplantation, this study conducted hydroponic lettuce transplanting every 7 days, with 30 trays and 360 seedlings planted each time. During the growth process of hydroponic lettuce, the nutrient solution in the cultivation tank was replaced every 2 days. The nutrient solution used for hydroponic lettuce was the Knop Classical Universal Hydroponic Formula, with specific ingredient content, as shown in Table 1.

### 2.2. Image Data Acquisition

**Figure 2:**
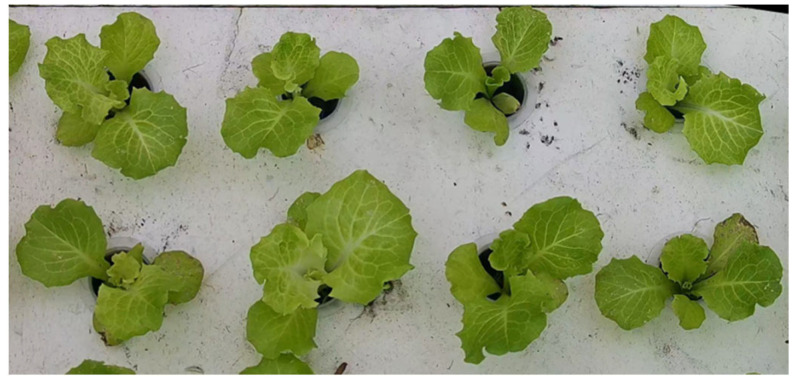
Video shooting effect.

After transplanting the hydroponic lettuce, videos of the lettuce growth were taken at fixed intervals every day during the following week. The shooting period was from 9:00 to 10:00 and from 15:00 to 16:00. The videos were recorded at a resolution of 2560 × 1440 and a frame rate of 30 fps. To ensure consistency in the collected image data, the camera was kept perpendicular to the tray surface at a distance of 60 cm. In total, four transplanting experiments were conducted and a total of 56 videos were recorded with a duration of 60 min each. Figure 2 shows the original video shooting effect.

### 2.3. Image Data Preprocessing

**Figure 3:**
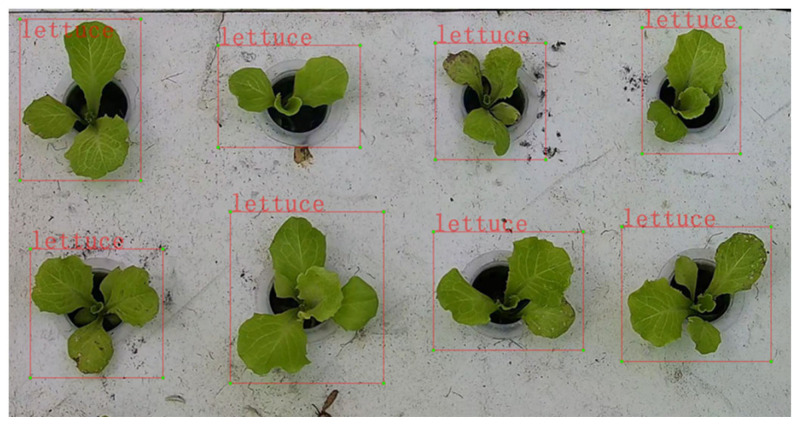
Annotated image of hydroponic lettuce in image dataset A.

Image data A: first, the captured lettuce videos were processed by using a video image algorithm. The algorithm was programed in Python and utilized the OpenCV and Random libraries. It was capable of batch processing video files and randomly extracted frame images from each video. A total of 40 images were randomly intercepted from each video, yielding a total of 2240 hydroponic lettuce images. Next, each lettuce image was labelled by using LabelImg 1.8.1 software and their labels were set to “lettuce” (as shown in Figure 3). Finally, the dataset was divided according to the ratio of 60% as training set, 20% as test set, and 20% as validation set.

**Figure 4:**
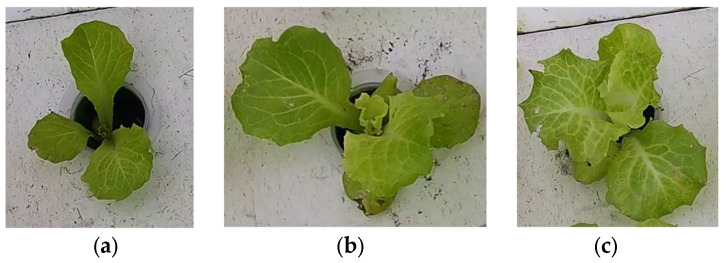
Images of hydroponic lettuce in different growth statuses from image dataset B. (**a**) Healthy lettuce. (**b**) Lettuce with diseases. (**c**) Lettuce with pests.

Image data B: first, the YOLO-v8n model was used to detect 56 hydroponic lettuce videos and extract individual hydroponic lettuce images from each frame of the videos. Due to limitations in GPU memory and training time considerations, a total of 12,000 randomly selected individual lettuce images were included in this dataset. The dataset consisted of images of hydroponic lettuce that depicted three different growth statuses, including healthy lettuce, lettuce with diseases, and lettuce infested with pests (as shown in Figure 4), with 6412, 2814, and 2774 images, respectively. Finally, the dataset was divided into training set, test set, and validation set according to a 3:1:1 ratio.

### 2.4. General Architecture of YOLO-EfficientNet

The recognition of the growth state of hydroponically grown lettuce faced several challenges, including strong background interference of lettuce growth, high similarity of lettuce leaves, and difficulty in classifying the growth state of lettuce. To solve these problems, this paper proposed a new method, namely YOLO-EfficientNet. The core idea of the YOLO-EfficientNet method was to combine the object detection model with the object classification model. The YOLO object detection model could accurately locate and identify the target objects in the image, while the EfficientNet object classification model could efficiently and accurately classify the target objects in the image. By combining the two, the YOLO-EfficientNet method could simultaneously achieve the localization and classification of target objects, thereby effectively solving the problem of difficulty in recognizing the growth state of hydroponically grown lettuce.

**Figure 5:**
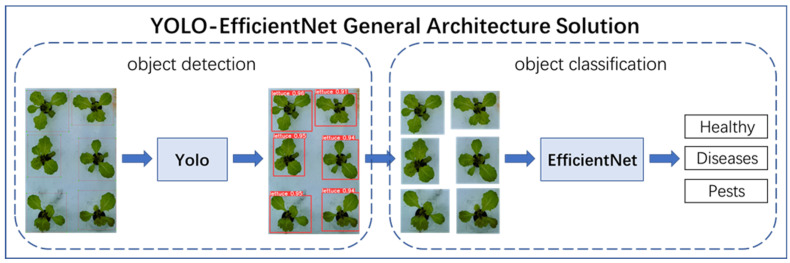
YOLO-EfficientNet model.

The overall architecture scheme is shown in Figure 5. The YOLO-EfficientNet method first used the YOLO object detection model to predict the hydroponic lettuce image, accurately marking the growth area of individual hydroponic lettuce with bounding boxes and cropping it. Then, the EfficientNet object classification model was used to classify the cropped images, distinguishing the three growth statuses of hydroponic lettuce: Healthy, Diseases, and Pests.

### 2.5. YOLO-v8 Model

**Figure 6:**
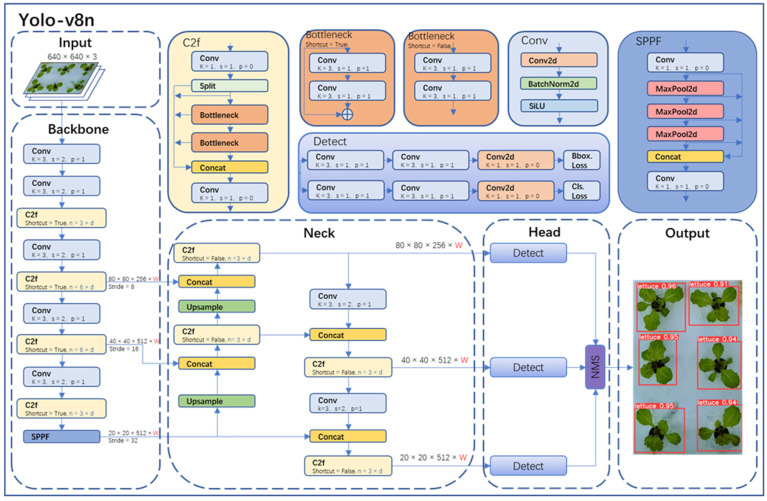
Yolo-v8n network structure.

The object detection part in this study adopted the state-of-the-art YOLO-v8 model from the YOLO series. YOLO-v8 was divided into YOLO-v8n, YOLO-v8s, YOLO-v8m, YOLO-v8l, and YOLO-v8x. Considering the model size, image resolution, GPU memory, and the requirement for accurate detection, this paper chose the YOLO-v8n network, which has a small size and high accuracy. The YOLO-v8n model detection network mainly consists of four parts (as shown in Figure 6): Input, Backbone, Neck, Head, and Output.

In the Input of YOLO-v8n, Mosaic technology was used for data augmentation of images, and this technology was turned off in the last 10 epochs of training. The specific operation of Mosaic technology was to combine four randomly selected images to create a new training sample, thereby increasing the diversity and richness of the data. Backbone in YOLO-v8n was primarily used for feature extraction. It consisted of modules like Conv, C2f, and spatial pyramid pooling fast (SPPF). In particular, the Conv module performed convolution, batch normalization (BN), and SiLU activation function operations on the input image. The C2f module was the main module for learning residual features, enabling YOLO-v8n to maintain rich gradient flow information while being lightweight. The SPPF module could transform feature maps of any size into fixed-size feature vectors.

Neck was primarily used for the fusion of multi-scale features to generate a feature pyramid. YOLO-v8n utilized the PANet structure as the core of its neck network. PANet consisted of two parts: feature pyramid network (FPN) and path aggregation network (PAN). FPN utilized a top-down pathway with up-sampling and fusion of coarser feature maps to achieve feature fusion across different levels. However, it lacked precise localization information of objects. PAN, on the other hand, used a bottom-up pathway with convolutional layers to fuse features from different levels, thus effectively preserving spatial information. The combination of FPN and PAN fully integrated the top-down and bottom-up information flow in the network, thereby improving the detection performance.

Head was the final prediction part of the model, used to obtain information about the class and location of objects of different sizes based on feature maps of different sizes. YOLO-v8n also used the non-maximum suppression (NMS) algorithm to further improve detection performance.

### 2.6. EfficientNet-v2 Model

**Figure 7:**
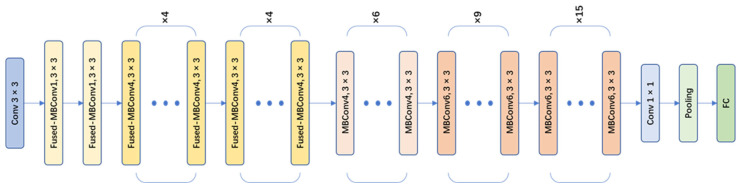
EfficientNet-v2s network architecture.

**Figure 8:**
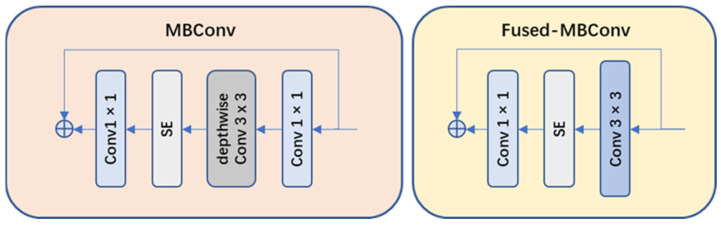
Architecture of the MBConv module and the Fused-MBConv module.

EfficientNet-v2 is a CNN model designed for image classification tasks. It is an improved and optimized version based on the EfficientNet series by Google [20]. The EfficientNet-v2 series includes EfficientNet-v2s, EfficientNet-v2m, Efficient-Net-v2l, and EfficientNet-v2xl. In this paper, the EfficientNet-v2s network (as shown in Figure 7), which has a small size and high accuracy, was chosen as the main network for object classification. The EfficientNet-v2s model incorporated a series of innovative network architecture designs and training strategies, further improving the recognition performance and training efficiency of the model. There were some improvements of EfficientNet-v2 compared to the v1 version:

(1) Network structure optimization: compared to v1, EfficientNet-v2 replaced the original strategy of equally scaling the models with a nonuniform scaling strategy to speed up model training. EfficientNet-v2 also introduced more diverse width and depth variations to adapt to different task requirements. Moreover, EfficientNet-v2 introduced MBConv and Fused-MBConv modules (as shown in Figure 8) to facilitate feature information transmission and communication.
(2) Training strategy optimization: EfficientNet-v2 introduced an improved progressive learning method, which dynamically adjusted the regularization methods based on the size of training images to enhance training speed and accuracy. Through experiments compared with some previous networks, the improved EfficientNet-v2 training speed was increased by 11 times and the number of training parameters was reduced to 1/7 of the original.

## 3. Results

### 3.1. Model Evaluation

The validation of model performance was crucial. True positive (*TP*) referred to the number of samples that were actually positive and predicted as positive in the sample set. True negative (*TN*) referred to the number of samples that were actually negative and predicted as negative in the sample set. False positive (*FP*) referred to the number of samples that were actually negative but predicted as positive in the sample set. False negative (*FN*) referred to the number of samples that were actually positive but predicted as negative in the sample set. Based on *TP*, *TN*, *FP*, and *FN*, various metrics were defined, including *Accuracy*, *Precision*, *Recall*, *mAP*, and *F_1_-Score*. The specific definitions were as follows:

*Accuracy*: It was defined as the ratio of correctly predicted samples to all samples.

(1) $$Accuracy=\frac{TP+TN}{TP+FN+FP+TN}$$

*Precision*: It was defined as the ratio of the number of samples correctly predicted as positive to the total number of samples predicted as positive.

(2) $$Precision=\frac{TP}{TP+FP}$$

*Recall*: It was defined as the ratio of the number of samples correctly predicted as positive to the total number of actual positive samples.

(3) $$Recall=\frac{TP}{TP+FN}$$

*F*_1_-*Score*: It was defined as the weighted average of Precision and Recall.

(4) $$F_{1}-Score=\frac{2\times Recall\times Precision}{Recall+Precision}$$

*mAP*: The term “*mAP*@0.5” referred to the average *precision* value when the IoU threshold was set to 0.5 for a particular class of samples. It reflected how the *precision* of the model changed with respect to *recall*. A higher value indicated that the model was more likely to maintain high *precision* at high *recall* rates. On the other hand, “*mAP*@0.5:0.95” represented the mean average *precision* across different IoU thresholds ranging from 0.5 to 0.95. The calculation method was as follows:

(5) $$AP@0.5=\frac{1}{n}\sum_{i=1}^{n}P_{i}=\frac{1}{n}p_{1}+\frac{1}{n}p_{2}+\cdots +\frac{1}{n}p_{n}$$

(6) $$mAP@0.5=\frac{1}{C}\sum_{k=1}^{C}AP@0.5_{k}$$

(7) $$mAP@0.5:0.95=\frac{1}{10}mAP@0.5+\frac{1}{10}mAP@0.55+\cdots +\frac{1}{10}mAP@0.95$$

### 3.2. Experimental Operation Environment

The experimental hardware environment for this study is shown in Table 2. In this project, Python 3.8 was used as the programing language and GPU acceleration was employed during model training using CUDA and CUDNN. Additionally, the deep learning framework PyTorch 1.13.1 was utilized for constructing the models.

### 3.3. Model Training Parameter

The training parameters for the object detection model in this study are shown in Table 3 and the training parameters for the object classification model are shown in Table 4.

### 3.4. Object Detection Results

**Figure 9:**
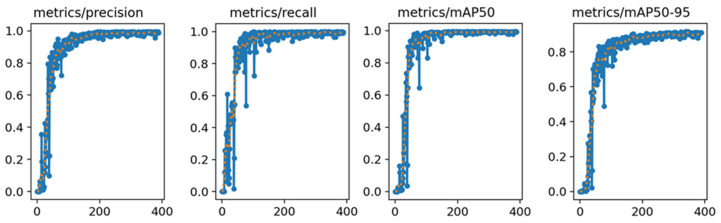
YOLO-v8n model training results. The blue line represents the actual data, while the orange line represents the data after smoothing.

The YOLO-v8n model was trained using the object detection model training parameters (as shown in Table 3) and used image dataset A as the training data for the model. The resulting training curve, as depicted in Figure 9, provided a visual representation of the model’s performance across the training epochs.

After carefully analyzing the training results, several key pieces of data were obtained. Notably, the YOLO-v8n model exhibited remarkable stability in its Precision and Recall rates, consistently achieving approximately 99% in both metrics for object detection tasks. This performance level indicated that the model had high accuracy in correctly identifying objects and was able to minimize the occurrence of misidentifying objects to the greatest extent. In addition to this, the model also demonstrated high competency in terms of the mAP, reaching a commendable level of 99%. The mAP was a crucial metric in object detection models, as it provided a comprehensive measure of the model’s precision and recall capabilities. The high mAP score indicated that the model was not only accurate but also reliable in its predictions.

**Figure 10:**
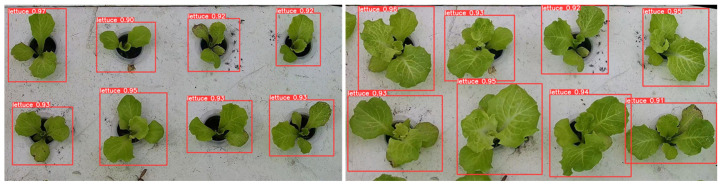
YOLO-v8n model object detection effect.

Upon evaluation, the conclusion was drawn that the YOLO-v8n model exhibited excellent performance in object detection tasks, particularly in the context of hydroponic lettuce targets. The final detection results not only met but exceeded the initial expectations set for the model. Figure 10 provided a visual demonstration of the model’s detection capabilities. The hydroponic lettuce in the image was accurately identified and encased within a rectangular bounding box, a standard practice in object detection tasks. Above the box, the confidence value of the model’s prediction was displayed, further demonstrating the model’s high level of certainty in its detection of the lettuce plant.

### 3.5. Object Classification Results

The EfficientNet-v2s model was trained using the object classification model training parameters (as shown in Table 4). In this experiment, image data B were used as the input for training the EfficientNet-v2s model. The training process was carried out for 250 epochs, which referred to the number of times the entire dataset was passed through the model during training. By training the model for a sufficient number of epochs, it led to improved classification accuracy. In order to evaluate the effectiveness of the EfficientNet-v2s model, a comparative analysis was conducted on six different deep learning network models. These models were selected to represent various architectures and object classification methods. The purpose of this comparison was to demonstrate the superior performance of our model compared to these existing models. To ensure fair and unbiased evaluation, all experiments were conducted under the same conditions. This included using the same training configuration, training strategy, and dataset. By keeping these factors consistent across all models, any differences in performance could be attributed to the architectural design and capabilities of the models themselves, rather than external factors.

Based on the results presented in Table 5, it was evident that EfficientNet-v2s model exhibited superior performance compared to the other six models across multiple evaluation metrics. These metrics included Val-acc (validation accuracy), Test-acc (test accuracy), Recall, Precision, and F1-score. In terms of Val-acc and Test-acc, the model of this paper achieved higher accuracy rates compared to the other models. This indicated that the EfficientNet-v2s model was more effective in correctly classifying objects during both validation and testing phases. Furthermore, the EfficientNet-v2s model demonstrated notable improvements over the best-performing DenseNet169 model among the other six models. Specifically, results showed a 3.54% increase in Val-acc, a 4.42% increase in Test-acc, a 7.9% increase in Recall, a 6.1% increase in Precision, and a 6.54% increase in F1-score. Through rigorous experimental setup and comparative analysis, strong support was provided for the superiority of the EfficientNet-v2s model over other testing models in terms of object classification accuracy and performance. This also demonstrated its effectiveness and application potential in the task of classifying images of diseases and pests in hydroponic lettuce in agriculture.

**Figure 11:**
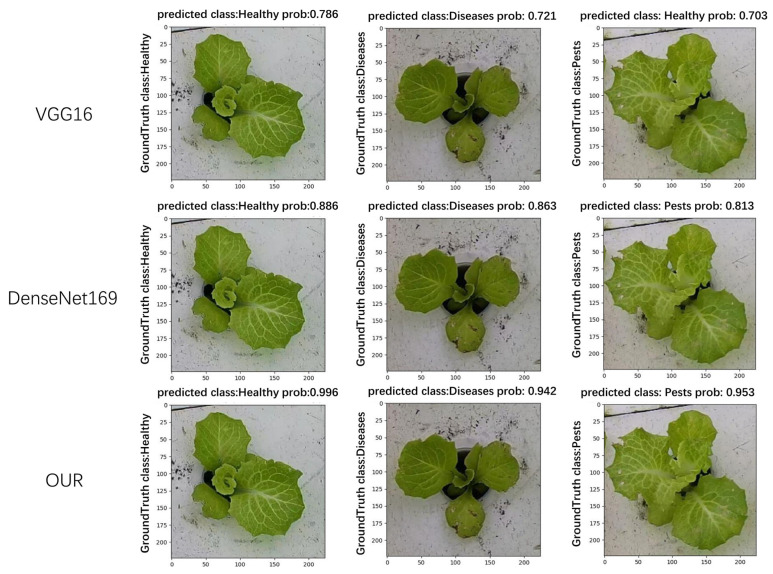
Comparison of model recognition effect.

In this paper, a comparison of the recognition performance of different models was also conducted. Each model was tasked with recognizing the growth status of hydroponic lettuce images. After recognition, the Ground Truth class, Predicted class, and Prob (Probability: score used to represent the predicted results of the image) for each image were outputted. As shown in Figure 11, it can be observed that VGG16 exhibited the worst recognition performance. Not only did it have low confidence scores but it also tended to misclassify lettuce with pests as healthy lettuce. The next model, DenseNet169, produced correct recognition results but its confidence scores were significantly lower compared to the model used in this paper.

Therefore, through analysis, YOLO-EfficientNet combined the advantages of object detection and object classification. It reduced the interference of growth background in lettuce images and increased the amount of training data for the object classification model, greatly improving the overall performance and effectiveness of model recognition.

## 4. Discussion

This study investigated the potential application of YOLO-EfficientNet in identifying the growth status in hydroponic lettuce. The results indicated that the model achieved a high level of accuracy in identifying the growth status. Compared to other recognition models, this method demonstrated higher accuracy and robustness. This was because this study leveraged the YOLO object detection model, which accurately located individual hydroponic lettuce images, and also utilized the advantages of the EfficientNet object classification model to more precisely determine whether the lettuce in hydroponic images had been affected by pests or diseases.

The results of this study were consistent with those of previous research. Phan et al. [21] proposed combining the Yolov5 with a convolutional neural network model in a deep learning framework, applied to classify tomatoes into three growth statuses: mature, immature, and damaged. This study demonstrated the effectiveness of using the YOLO-EfficientNet method, which combines the object detection model with the object classification model, in image classification tasks. Furthermore, a study also explored the potential practical application of semantic segmentation models in the machine sorting system for harvesting hydroponic lettuce. Wu et al. [22] utilized the DeepLabV3+ model with four different backbones (ResNet-50, ResNet-101, Xception-65, and Xception-71) to develop a visual segmentation system for identifying abnormal hydroponic lettuce leaves (yellowing, withering, and rotting). This method enabled the rapid removal of abnormal leaves from hydroponic lettuce, reducing manual sorting costs, extending the shelf life of hydroponic lettuce, and increasing its market value.

Although the application of image recognition technology in agriculture had tremendous potential, it was necessary to acknowledge the limitations of this study. These included the need for further validation of the method’s recognition performance under different environmental conditions. Testing the model on various lettuce varieties and even different types of crops was essential to evaluate the effectiveness and generalizability of this approach. Future research should focus on addressing these limitations and exploring the integration of image recognition models with automated monitoring systems to provide timely and accurate management recommendations for growers.

## 5. Conclusions

This paper addressed the issue of pest and disease detection in hydroponic lettuce after transplanting and proposed a method called YOLO-EfficientNet for identifying the growth status of hydroponic lettuce. The YOLO-v8n network was used to crop and segment each hydroponic lettuce plant, reducing the interference of environmental backgrounds in lettuce images. The segmented dataset provided sufficient training data for the EfficientNet-v2s model. In the recognition results of YOLO-EfficientNet, excellent scores were achieved in Val-acc, Test-acc, Recall, Precision, and F1-score, with values of 95.78, 94.68, 96.02, 96.32, and 96.18, respectively, surpassing other recognition models. YOLO-EfficientNet simplified the problem of group classification into individual classification, overcoming challenges such as similarity among small object crops and background interference, thus improving the accuracy of object detection. Furthermore, the YOLO-EfficientNet model effectively handled limited sample data during training, partially addressing the issue of insufficient data.

In conclusion, the application of the YOLO-EfficientNet method significantly improved the performance of object recognition in hydroponic lettuce, providing accurate and efficient detection of the growth status.

## Figures and Tables

**Table 1:** Knop Classical Universal Hydroponic Formula.

Ingredient Name	Volume (mg/L)
Ca(NO_3_)_2_·4H_2_O	1150
KNO_3_	200
MgSO_4_·7H_2_O	200
KH_2_PO_4_	200
Total salt content	1750

**Table 2:** Configuration of experimental hardware environment.

Hardware	Model	Number
Main board	MAG B660M MORTAR WIFI DDR4	1
CPU	i5-12490F	1
GPU	RTX 3070	2
Memory	32 GB	2
Solid state drives	WDC WD20EZBX-00AYRAO(2 T)	1
Hard disk	WD Blue SN570 500GB SSD (500 GB) Great Wall GT70 1 TB (1 TB)	1

**Table 3:** Object detection model training parameter.

Parameter	Value	Parameter	Value
Optimizer	SGD	Lrf	0.01
Epochs	400	Weight decay	0.0005
Batch size	16	momentum	0.937
Workers	16	Warmup epochs	3
Image size	640 × 640	Warmup momentum	8
Lr0	0.001	Close mosaic	10

**Table 4:** Object classification model training parameter.

Parameter	Value
Optimization	Adam
Learning rate	0.0001
Epochs	250
Batch size	16
Workers	16
Image size	224 × 224

**Table 5:** Recognition results of various object classification models.

Models	Train-acc/%	Val-acc/%	Test-acc/%	Recall/%	Precision/%	F1-Score/%
VGG16	91.43	86.32	78.48	80.72	81.62	81.12
ResNet50	97.34	90.82	88.34	86.88	88.02	87.14
GoogleNet	99.21	92.24	90.26	88.12	90.22	89.64
DenseNet169	99.72	93.42	89.14	90.24	91.24	90.72
MobileNet-v1	94.46	88.l8	84.28	84.68	85.24	84.92
MobileNet-v2	96.31	90.54	88.42	88.72	89.48	89.10
ShuffleNet-v1	93.68	88.26	83.18	84.72	86.02	85.36
ShuffleNet-v2	95.18	92.58	90.46	89.42	90.48	89.92
ours	99.32	**95.78**	**94.68**	**96.02**	**96.32**	**96.18**

## Data Availability

The dataset publicly available on Kaggle at https://www.kaggle.com/datasets/wingsdong/lettuce-diseases-and-pests/data.
